# Supplementary material for: Oncometabolite D-2-hydroxyglutarate—dependent metabolic reprogramming induces skeletal muscle atrophy during cancer cachexia
Source: Commun Biol. 2023 Sep 23;6:977. doi: 10.1038/s42003-023-05366-0 (PMC10518016; doi:10.1038/s42003-023-05366-0)
Supplement: Supplementary file 2 — Supplementary Materials [file 42003_2023_5366_MOESM2_ESM.pdf]

Oncometabolite D-2-hydroxyglutarate - dependent metabolic reprogramming induces skeletal muscle atrophy during cancer cachexia

Xinting Zhu[a*], Juan Hao[b*], Hong Zhang[a], Mengyi Chi[a], Yaxian Wang[a], Jinlu Huang[a], Rong Xu[a], Xincai, Zhao[a], Bo Xin[a], Xipeng Sun[a], Jianping Zhang[a], Shumin Zhou[c], Dongdong Cheng[d], Ting Yuan[d], Jun Ding[e], Shuier Zheng[f], Cheng Guo[a#], Quanjun Yang[a#]

[a] Department of Pharmacy, Shanghai Sixth People's Hospital affiliated Shanghai Jiao Tong University School of Medicine, 600 Yishan Road, Shanghai 200233, China

[b] Department of Endocrinology, Shanghai Traditional Chinese Medicine, Integrated Hospital, Shanghai University of Traditional Chinese Medicine, 230 Baoding Road, Shanghai 200082, China

[c]Institution of microsurgery on extremities, Shanghai Sixth People's Hospital affiliated Shanghai Jiao Tong University School of Medicine, 600 Yishan Road, Shanghai 200233, China

[d,] Department of Bone Oncology, Shanghai Sixth People's Hospital affiliated Shanghai Jiao Tong University School of MedicineShanghai , P. R. China.

[e] Department of Neurosurgery, Shanghai Sixth People's Hospital affiliated Shanghai Jiao Tong University School of Medicine, 600 Yishan Road, Shanghai 200233, China

[f]Department of Oncology, Shanghai Sixth People's Hospital affiliated Shanghai Jiao Tong University School of Medicine, 600 Yishan Road, Shanghai 200233, China

**These authors contributed equally to this work*

[#] Corresponding Author:

Quanjun Yang, myotime@sjtu.edu.cn

Cheng Guo, guopharm@126.com

Supplementary Figure S1. The gene expression profiles in all tumor samples/paired normal tissues and the mutation profile of *IDH1* transcript.

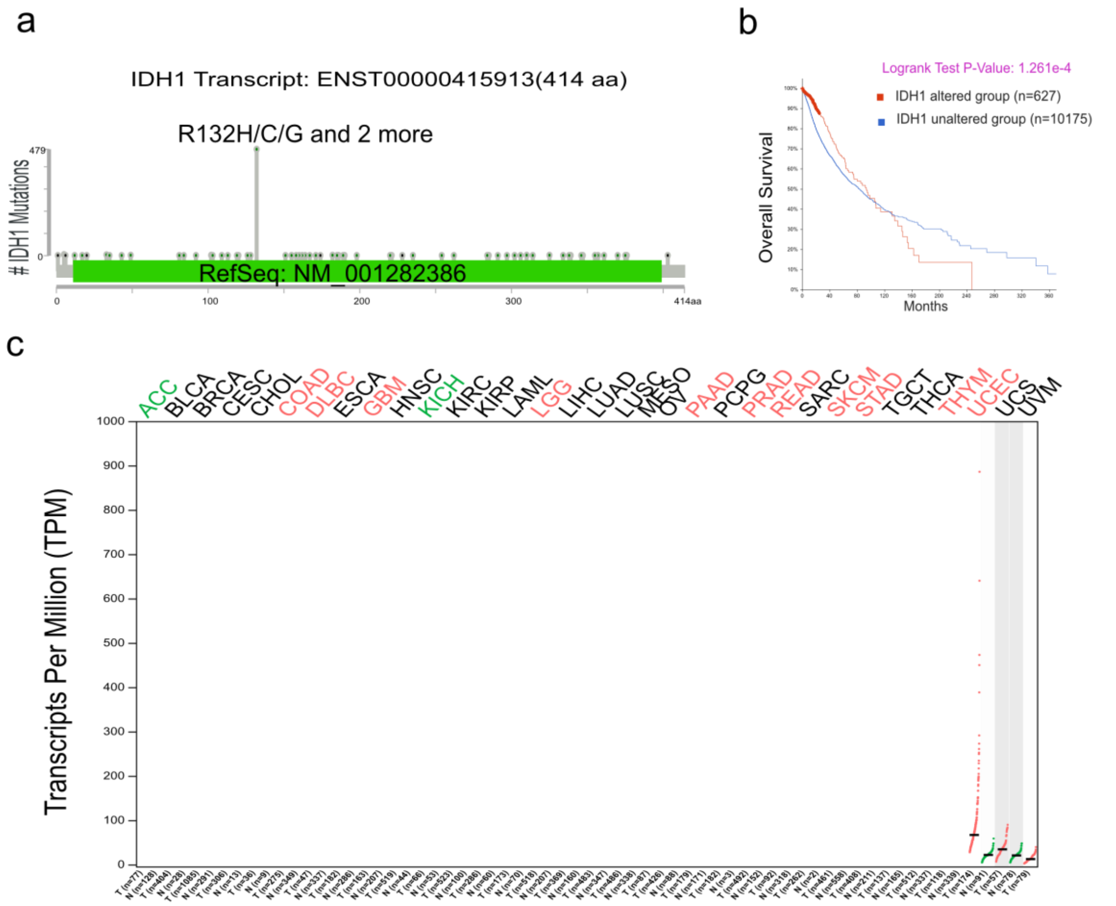

a *IDH1* transcript mutation profile. b Overall survival of *IDH1* mutation (n=627) and *IDH1* unaltered groups (n=10,175) with pan-cancer types from The Cancer Genome Atlas. Data were catalyzed from the cBioportal portal (https://www.cbioportal.org/). c The gene expression profile across all tumor samples and paired normal tissues. Each point represents the expression of one sample.

Supplementary Figure S2. The *IDH1* vector used for animal studies.

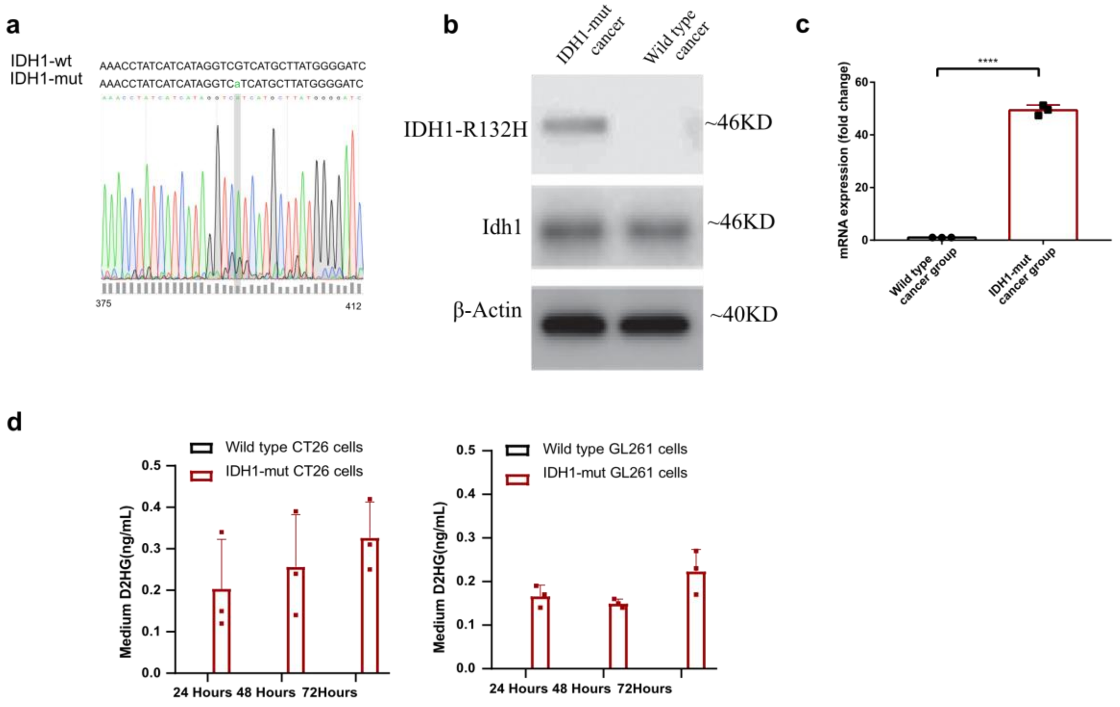

a The sequence of *IDH1* (R132H) mutation at the position of rs121913500. b Western blot of *IDH1* (R132H) mutation and wild-type *IDH1* protein expression in *IDH1* mutant cancer and wild-type cancer groups. c Column plot of *IDH1* mRNA expression in *IDH1* mutant cancer group and wild-type cancer group. Unpaired two-tailed t-test (****p<1e-4, ***p<2e-4, **p<2e-3, *p<0.05). d Column plot of mean D2HG levels in wild-type and *IDH1* mutant cancer cells of CT26 and GL261 cells.

Supplementary Figure S3. Uncropped pictures of western blotting membranes

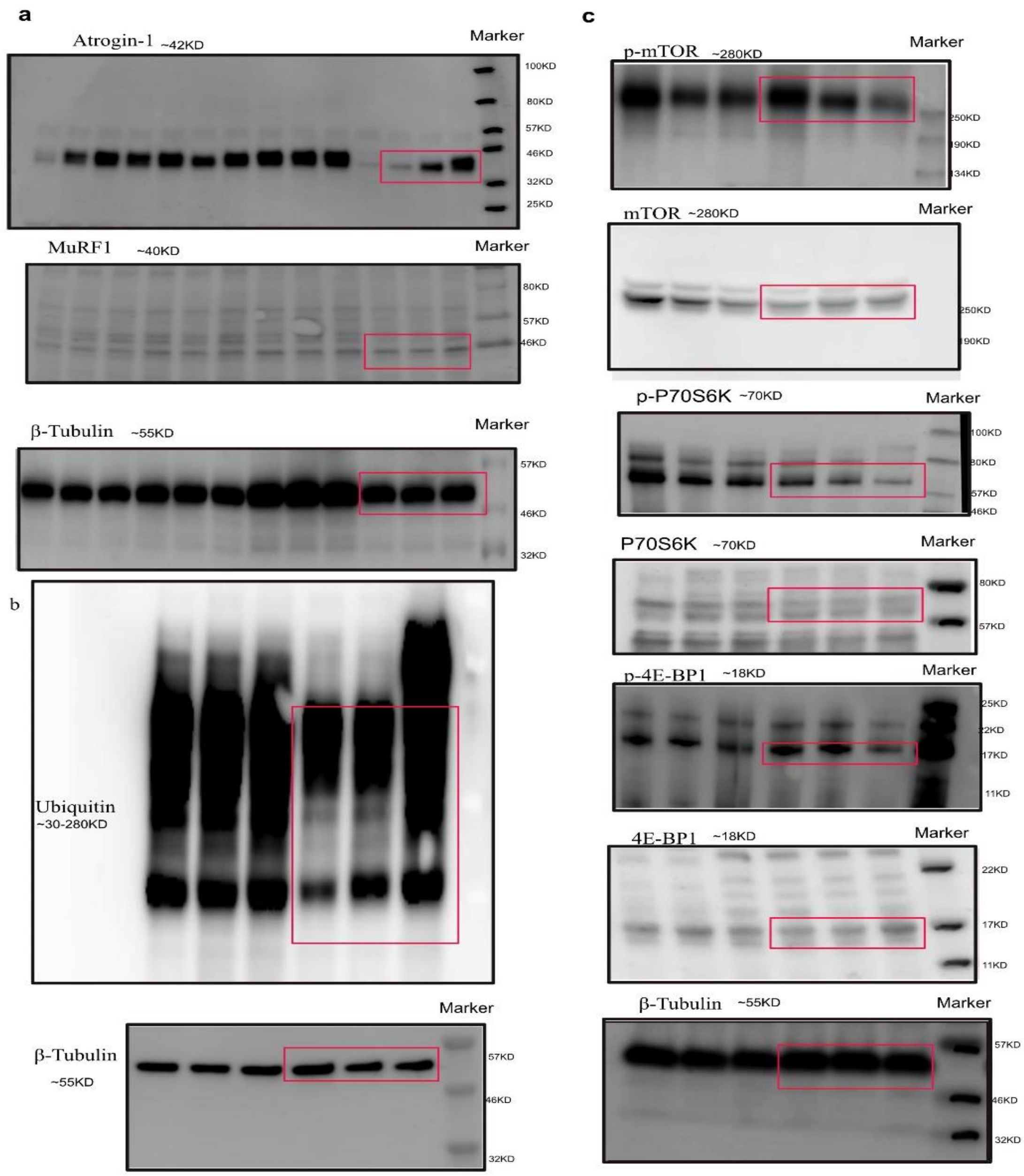

a The protein expression of the degradation-related E3 ligases atrogin-1 and MuRF1 in *IDH1*-mutant cancer-bearing mice and wild-type cancer-bearing mice (Original blot of Fig 3b). b The protein expression of ubiquitin in *IDH1* mutant cancer-bearing mice and wild-type cancer-bearing mice (Original blot of Fig 3d). c The protein expression of synthesis-related mTOR, P70S6K and 4E-BP1 expression and phosphorylation levels in *IDH1* mutant cancer-bearing mice and wild-type cancer-bearing mice (Original blot of Fig 3e).

Supplementary Figure S4. D2HG-induced muscle wasting.

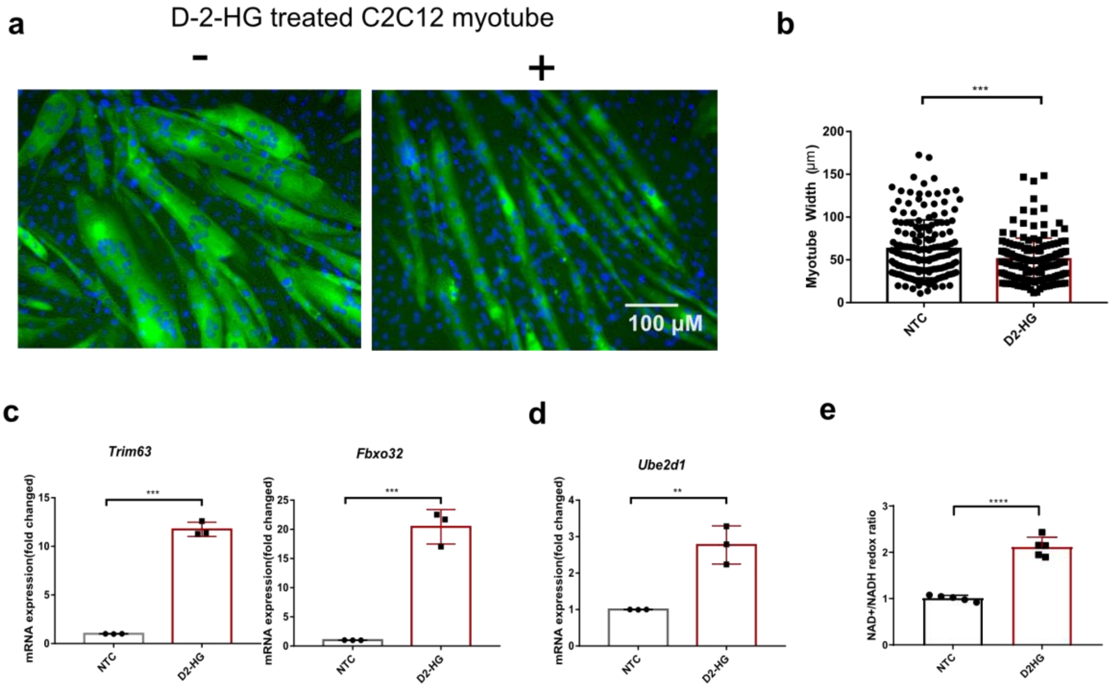

a Typical immunofluorescence morphological changes after D2HG treatment. b Column plot of myotube width in NTC- and D2HG-treated well-differentiated myotubes. Unpaired two-tailed t-test (****p<1e-4, ***p<2e-4, **p<2e-3, *p<0.05). c Column plot of mRNA expression of the E3 ligases Trim63 and Fbxo32. Unpaired two-tailed t-test (****p<1e-4, ***p<2e-4, **p<2e-3, *p<0.05). d Column plot of mRNA expression of the E2 ubiquitin conjugating enzyme Ube2d1. Unpaired two-tailed t-test (****p<1e-4, ***p<2e-4, **p<2e-3, *p<0.05). e Column plot of NAD+/NADH redox ratio in NTC- and D2HG-treated myotubes for 24 hours. Unpaired two-tailed t-test (****p<1e-4, ***p<2e-4, **p<2e-3, *p<0.05).

Supplementary Figure S5. The significantly altered pathways and gene sets between D2HG and NTC-treated well-differentiated myotubes.

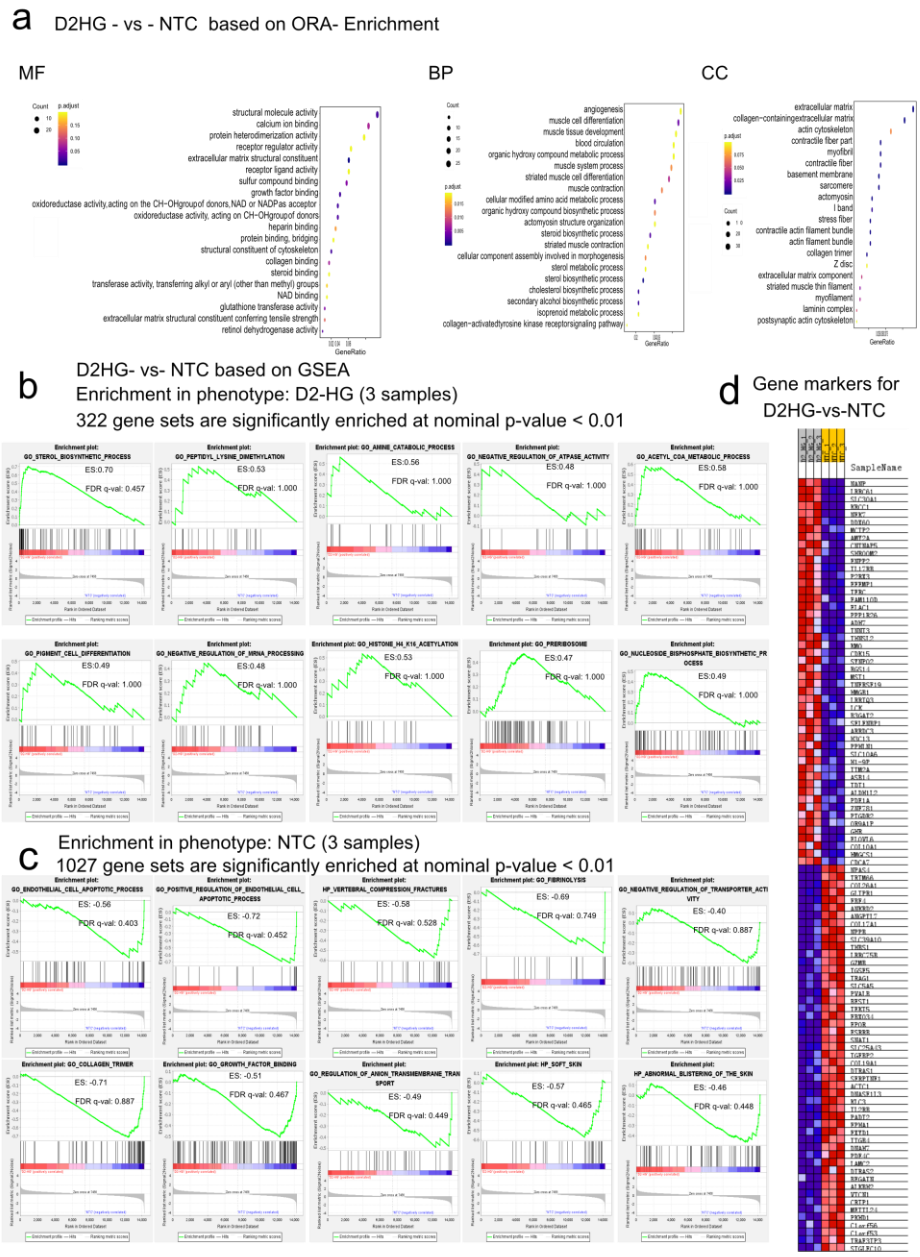

a Gene Ontology analysis revealed the molecular function, biological process, and cellular component between D2HG and NTC-treated well-differentiated myotubes. b Gene set enrichment analysis of significant gene sets enriched in D2HG-treated well-differentiated myotubes. c Gene set enrichment analysis of significant gene sets enriched in NTC-treated well-differentiated myotubes. d Heat map and gene list correlation profile for all features in the dataset.

Supplementary Figure S6 Uncropped pictures of western blotting membranes

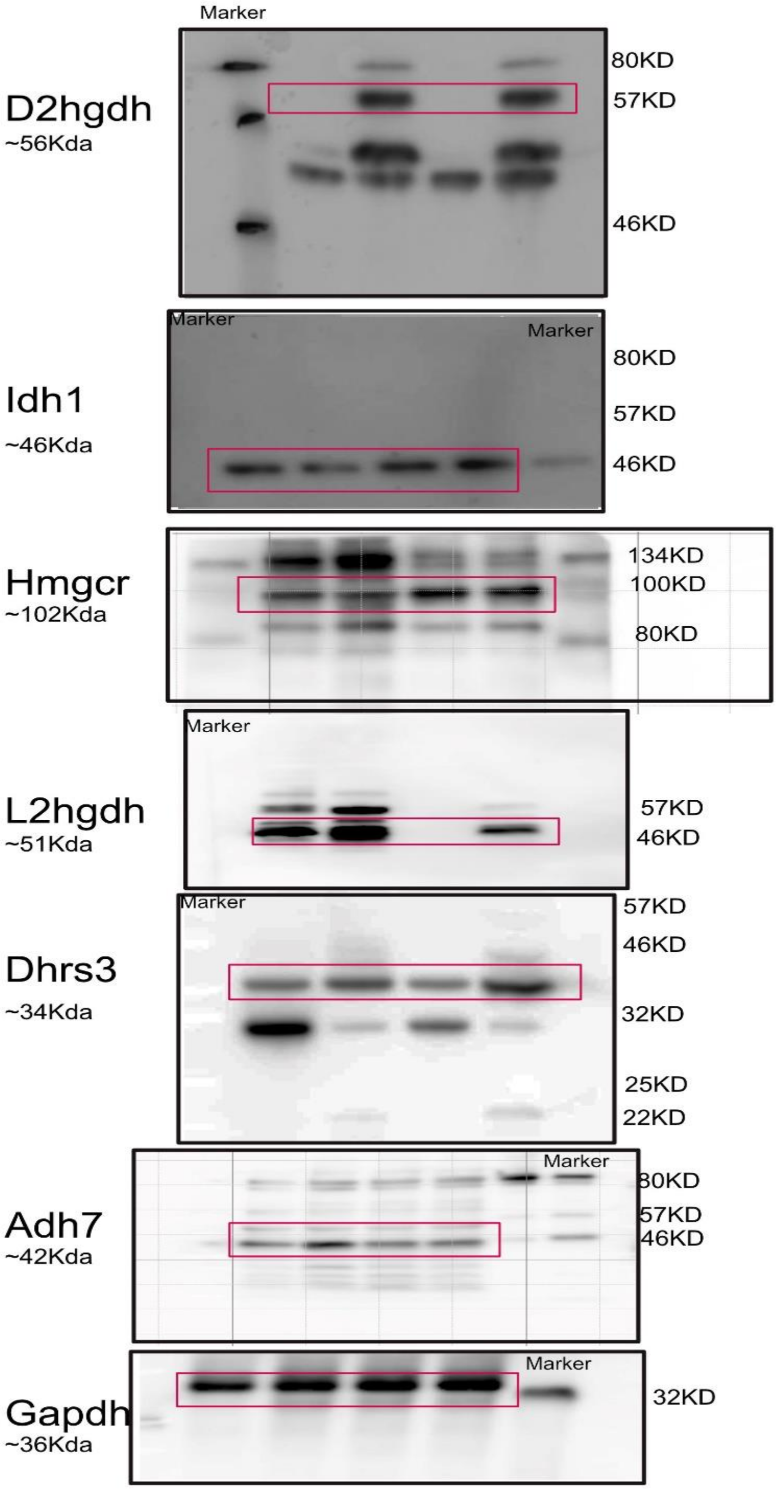

Western blot results of protein expression in NTC and D2HG-treated well-differentiated myotubes with/without *D2hgdh* overexpression. GAPDH was used as an internal reference.

Supplementary Figure S7. Metabolic pathway and relative levels of metabolites in D2HG-treated myotubes overexpressing *D2HGDH*.

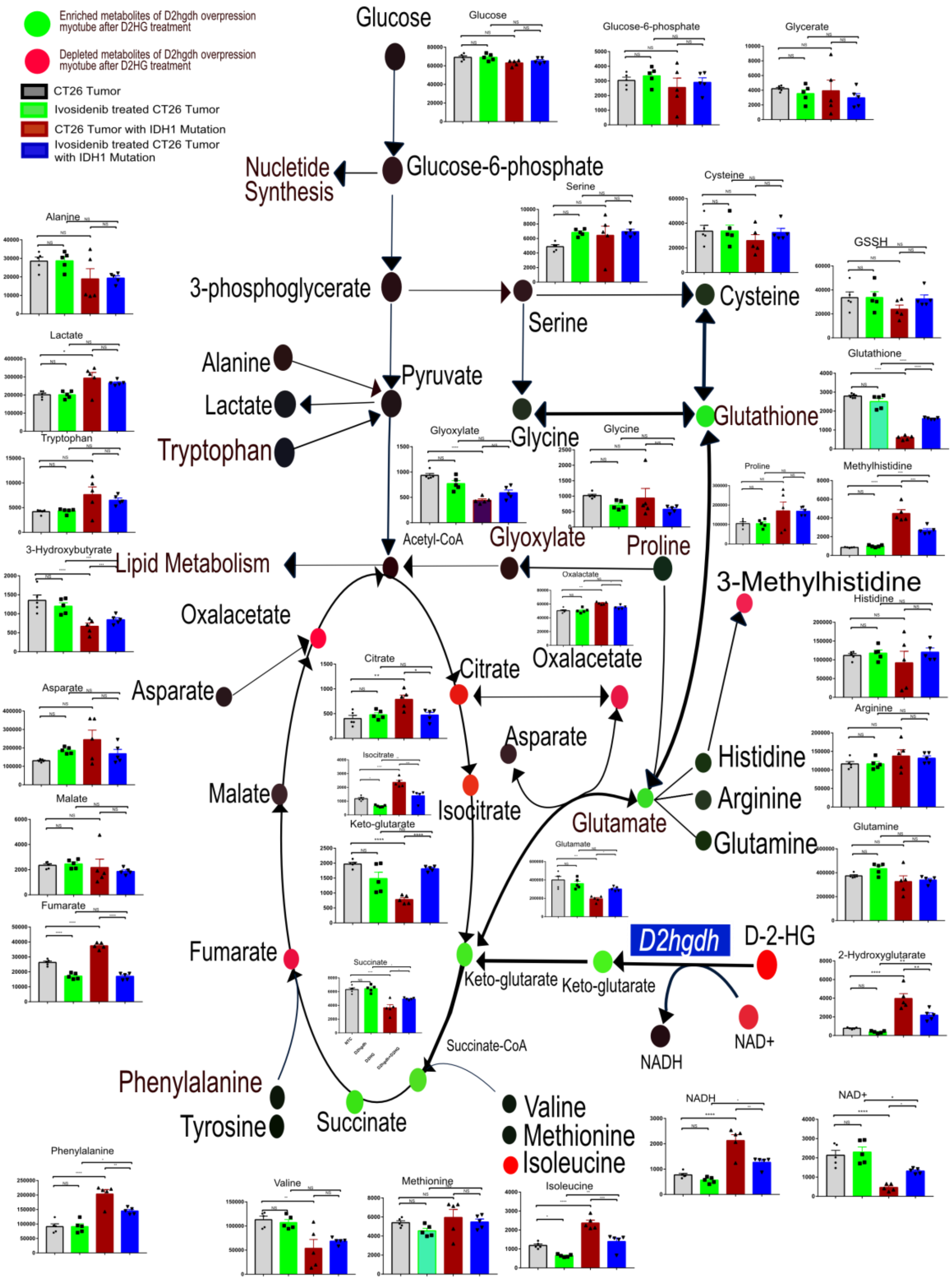

Supplementary Figure S8. The significantly altered pathways and gene sets between *D2HGDH* overexpression well-differentiated myotubes and NTC-treated well-differentiated myotubes.

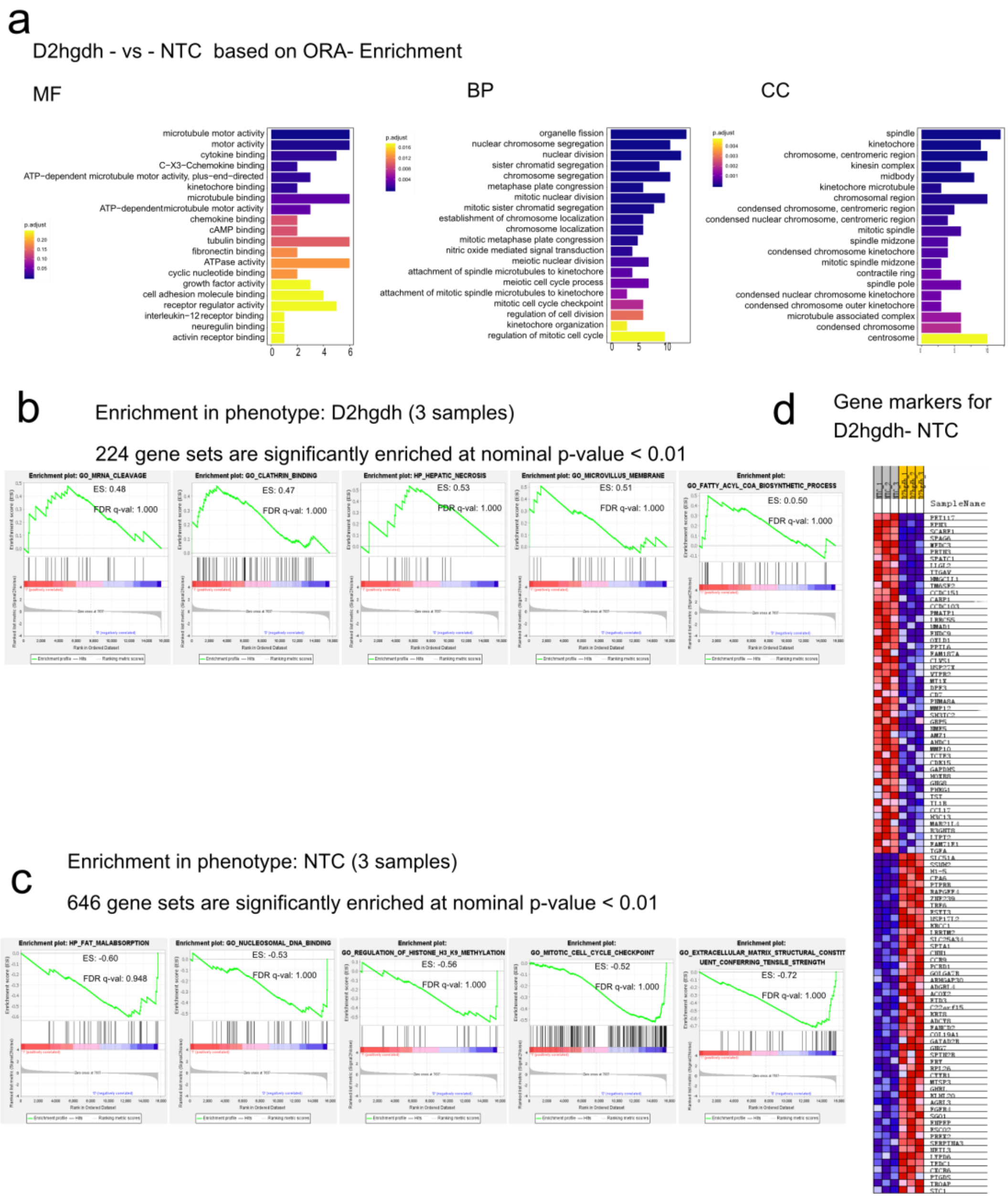

a Gene Ontology analysis revealed the molecular function, biological process, and cellular component between *D2HGDH* overexpression well-differentiated myotubes and NTC treated well-differentiated myotubes. b Gene set enrichment analysis of significant gene sets enriched in *D2HGDH*-overexpressing well-differentiated myotubes. c Gene set enrichment analysis of significant gene sets enriched in *D2HGDH* over-expressing well-differentiated myotubes. d Heat map and gene list correlation profile for all features in the dataset.

Supplementary Figure S9. The significantly altered pathways and gene sets between D2HG-treated well-differentiated *D2HGDH* overexpressing myotubes and NTC-treated well-differentiated *D2HGDH* overexpressing myotubes.

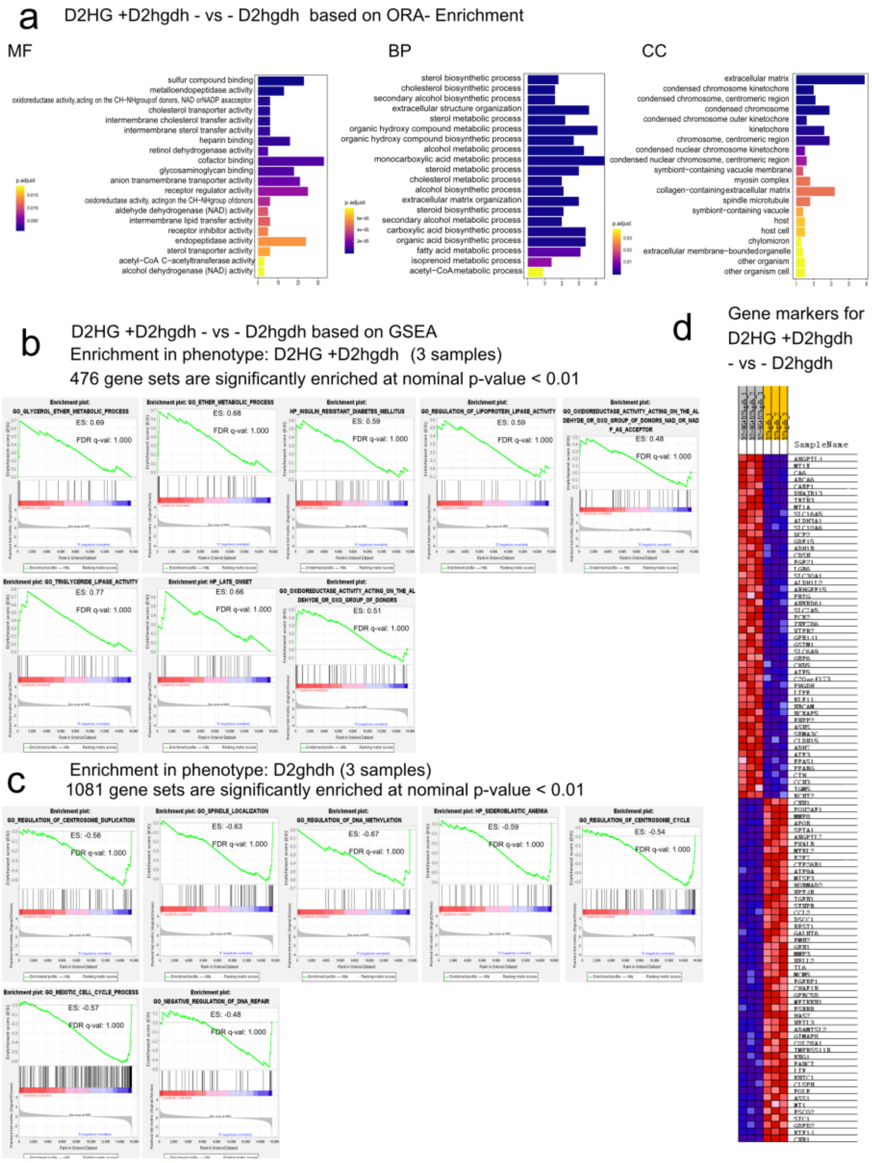

a Gene Ontology analysis revealed the molecular function, biological process, and cellular component between D2HG-treated *D2HGDH* overexpression well-differentiated myotubes and NTC-treated *D2HGDH* overexpression well-differentiated myotubes. bGene set enrichment analysis of significant gene sets enriched in D2HG-treated *D2HGDH* overexpressing well-differentiated myotubes. c Gene set enrichment analysis of significant gene sets enriched in NTC-treated *D2HGDH* overexpression well-differentiated myotubes. d Heat map and gene list correlation profile of all features in the dataset.

Supplementary Figure S10. The significantly altered pathways and gene sets between D2HG-treated *D2HGDH* overexpressing well-differentiated myotubes and D2HG-treated well-differentiated myotubes.

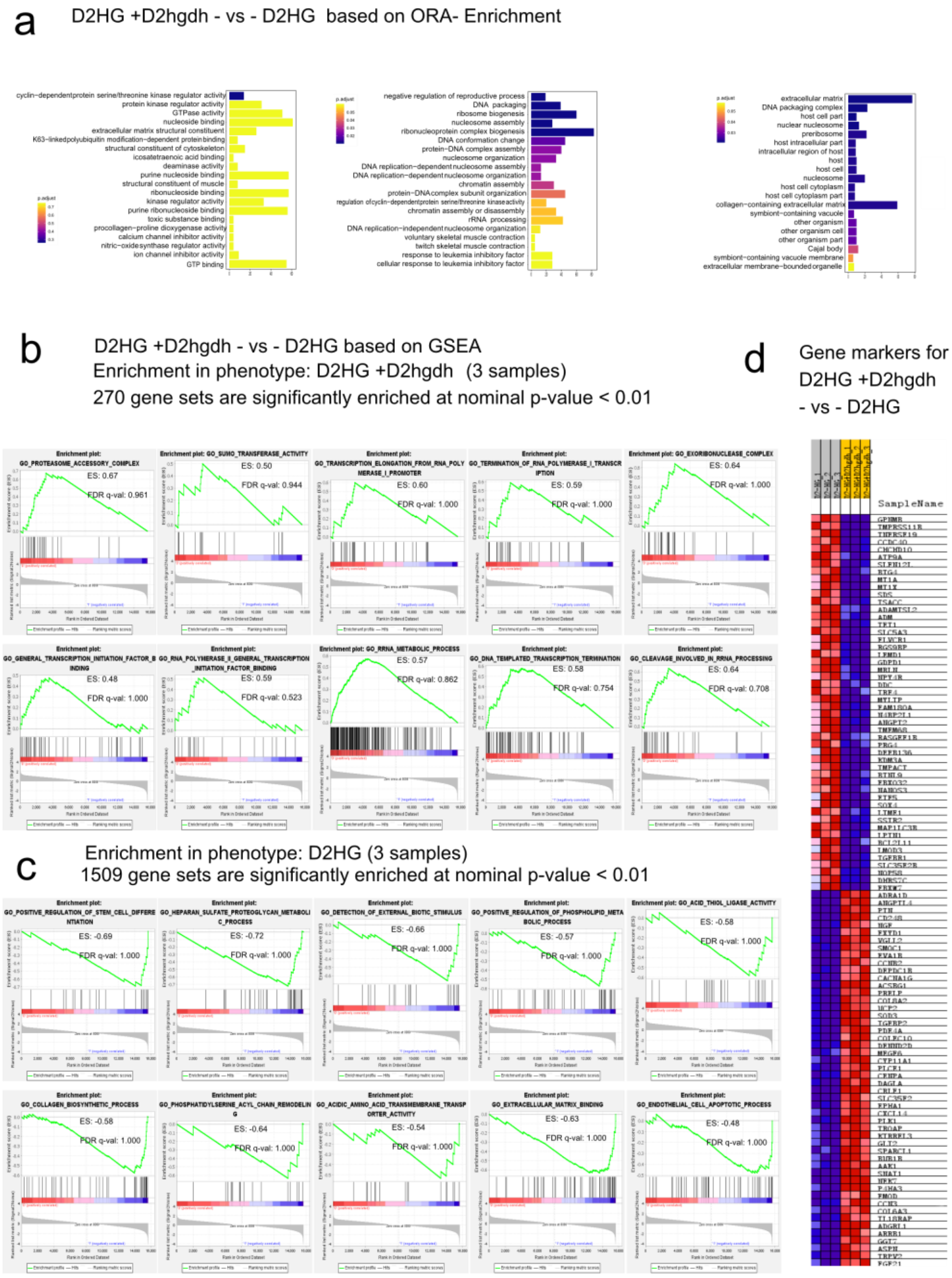

a Gene Ontology analysis revealed the molecular function, biological process, and cellular component between D2HG-treated *D2HGDH* overexpressing well-differentiated myotubes and ND2HG-treated well-differentiated myotubes. b Gene set enrichment analysis of significant gene sets enriched in D2HG-treated *D2HGDH* overexpressing well-differentiated myotubes. c Gene set enrichment analysis of significant gene sets enriched in D2HG-treated well-differentiated myotubes. d Heat map and gene list correlation profile of all features in the dataset.

Supplementary Figure S11. The mRNA expression of *Ube2d1, Trim63,* and *Fbxo32* in *IDH1*(R132H) mutant CT26 cancer and wild-type cancer treated with ivosidenib or NTC. Each group had three biological replicates.

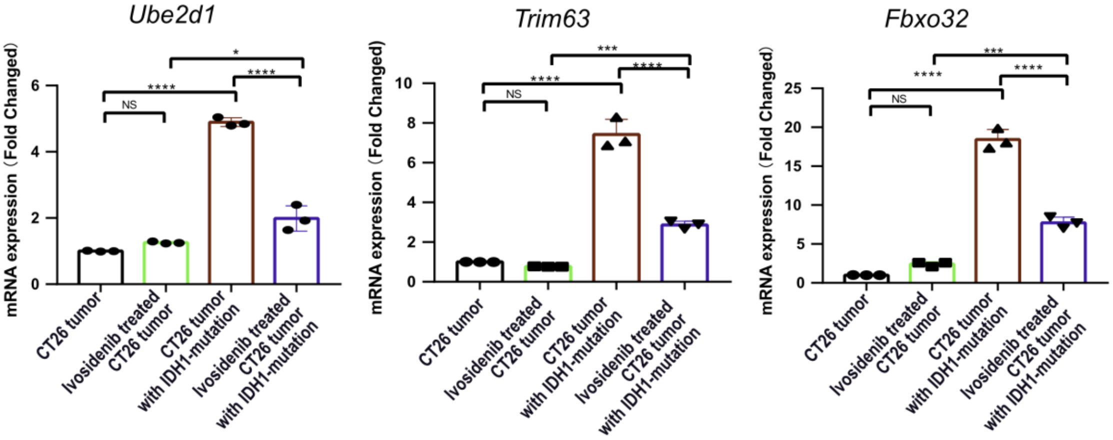

One-way ANOVA with post hoc Tukey's multiple comparison test (****p<1e-4, ***p<2e-4, **p<2e-3, *p<0.05).

Supplementary Figure S12. The change of body weight, gastrocnemius muscle and serum D2HG in *IDH1*(R132H) mutant GL261 cancer and wild-type GL261 cancer treated with ivosidenib or NTC.

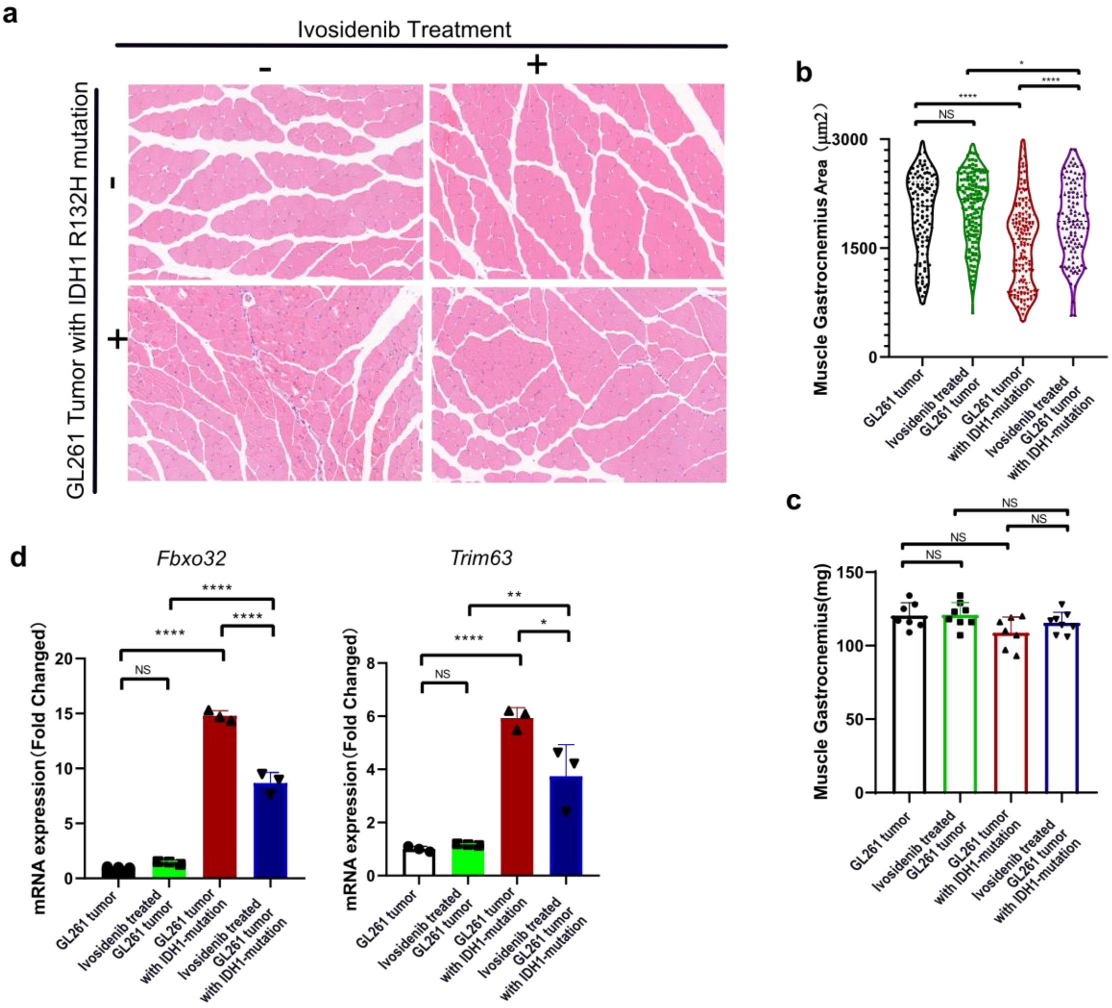

a Typical cross-sectional histopathologic image of gastrocnemius muscle in *IDH1* (R132H) mutant cancer and wild-type cancer treated with ivosidenib or NTC (scale bar: 50μm). b Column plot of gastrocnemius muscle cross-sectional area in *IDH1* (R132H) mutant cancer and wild-type cancer treated with ivosidenib or NTC. One-way ANOVA with post hoc Tukey's multiple comparison test (****p<1e-4, ***p<2e-4, **p<2e-3, *p<0.05). c Muscle gastrocnemius weight of mice in *IDH1* (R132H) mutant cancer group and wild-type cancer group treated with ivosidenib or NTC. d Column plot of mRNA expression of *Fbxo32* and *Trim63* in *IDH1* (R132H) mutant cancer group and wild-type cancer group treated with ivosidenib or NTC. One-way ANOVA with post hoc Tukey's multiple comparison test (****p<1e-4, ***p<2e-4, **p<2e-3, *p<0.05).

Supplementary Figure S13. The protein synthesis/degradation of gastrocnemius muscle in *IDH1*(R132H) mutant GL261 cancer and wild-type GL261 cancer treated with ivosidenib or NTC.

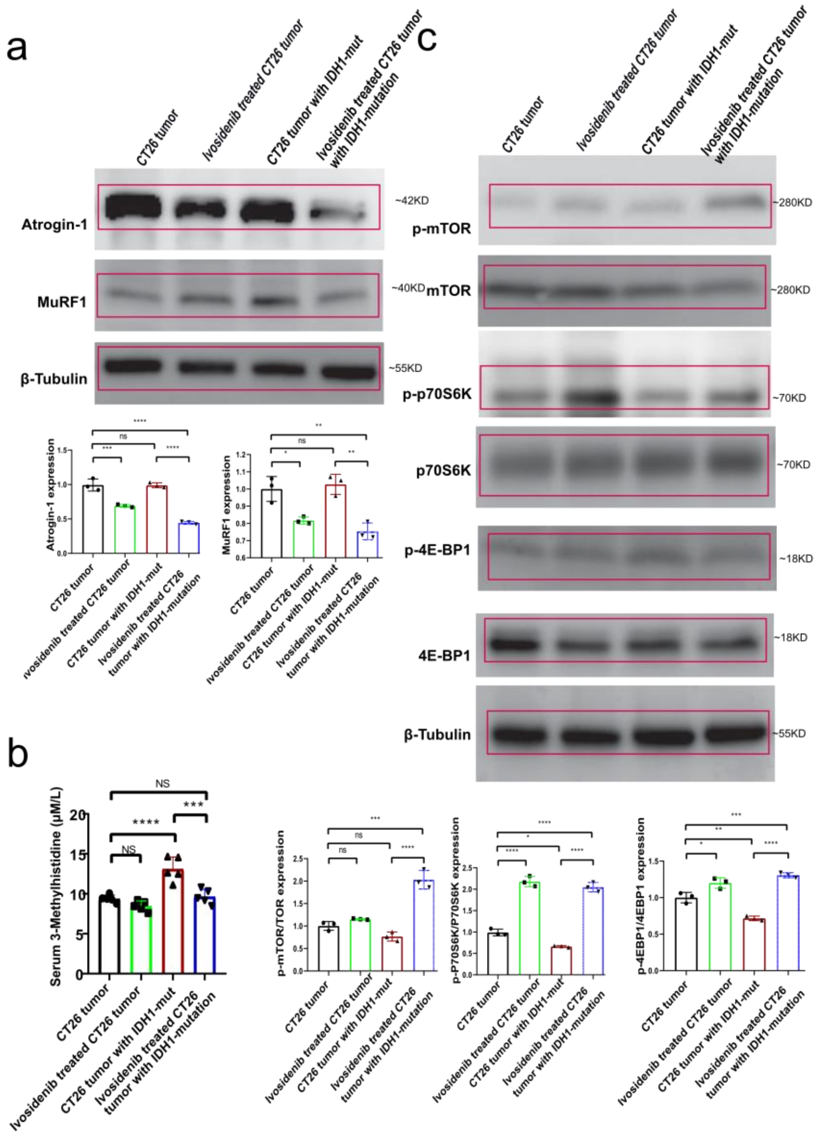

a The protein expression and quantified data of E3 ligase atrogin-1 and MuRF1. One-way ANOVA with post hoc Tukey's multiple comparison test (****p<1e-4, ***p<2e-4, **p<2e-3, *p<0.05). b Serum 3-methylhistidine levels. One-way ANOVA with post hoc Tukey's multiple comparison test (****p<1e-4, ***p<2e-4, **p<2e-3, *p<0.05). c The phosphorylation levels and quantified data of mTOR, p70s6k and 4E-BP1. One-way ANOVA with post hoc Tukey's multiple comparison test (****p<1e-4, ***p<2e-4, **p<2e-3, *p<0.05).

Supplementary Note

Materials

Metabolites were purchased as follows: disodium succinate (W327700), sodium fumarate dibasic (F1506), sodium L-lactate (L7022), D-2-hydroxyglutarate disodium salt (H8378), sodium pyruvate (P2256), adenosine (A4036), inosine (I4125), carnosine (C9625), phenylacetate (108723), phenylalanine (P2126), 1-methylhistidine (67520), 3-methylhistidine (M9005), 4-hydroxyproline (8.16007), 4-hydroxybutyrate (G-001), creatine (C3630), tryptophan (T9753), tyrosine (T3754), methionine (M9375), glutamate (1446600). Amino acid standards for protein hydrolysates (A9781) were from Sigma. Drugs were from Selleck and Sigma, such as dexamethasone (D4902, Sigma), ivosidenib (AG-120, S8206, Selleck), vorasidenib (S8611, Selleck), AGI-6780 (S7241, Selleck), BAY 1436032 (S8530, Selleck). Antibodies were from: anti-beta-actin antibody (AA128, Beyotime), anti-ADH7 antibody (ab186408, Abcam), anti-DHRS3 antibody (NBP1-80846, Novusbio), anti-GAPDH antibody (AF0006, Beyotime), anti-HMGCR antibody (ab174830, Abcam), anti-HSD17B7 antibody (16925-1-AP, Proteintech), anti-*IDH1* antibody (ab172964, Abcam), anti-*IDH1* antibody (ab230949, Abcam), anti-*IDH1* antibody (ab113232, Abcam), anti-*IDH1* (R132H) antibody (ab198123, Abcam), anti-*IDH1* (R132H) antibody (DIA-H09, Dianovo), anti-*IDH1* (R132H) antibody (SAB4200548, Sigma), anti-MYH antibody (sc-376157, SCBT), anti-MYH1/2/3 antibody (sc-53092, SCBT), and anti-skeletal muscle antibody (sc-32733, SCBT). Enzymes were from NEB: DNase I (RNase-Free) (M0303S, NEB), endonuclease AgeI-HF (R3552S, NEB), endonuclease BsmBI-v2(r0739s, NEB), endonuclease EcoRI-HF (R3101S, NEB), M-MuLV reverse transcriptase (M0253S, NEB), NEBuilder (E2621L, NEB), and Q5 High-Fidelity DNA polymerase (M0491L, NEB). The columns used for the separation of metabolites mainly from Phenomenex, listed as, Chirex 3126, LC Column 150 x 4.6 mm (00F-3126-E0,), Kinetex 2.6 µm PS C18, LC Column 150 x 2.1mm (00B-4780-E0, Phenomenex), SecurityGuard ULTRA Holder (AJ0-9000, Phenomenex), and SecurityGuard™ ULTRA Cartridges (AJ0-9532, Phenomenex). Cell CT26.wt colon carcinoma cell (CRL-2638) was from ATCC. GL261 (CTCC-007), HEK293 (GNHu 43) and C2C12 murine myoblast (SCSP-505) were from the Cell Bank of the Typical Culture Preservation Committee of the Chinese Academy of Sciences.
