# Supplementary material for: Oncometabolite D-2-hydroxyglutarate—dependent metabolic reprogramming induces skeletal muscle atrophy during cancer cachexia
Source: Commun Biol. 2023 Sep 23;6:977. doi: 10.1038/s42003-023-05366-0 (PMC10518016; doi:10.1038/s42003-023-05366-0)
Supplement: Supplementary file 3 — Description of Additional Supplementary Files [file 42003_2023_5366_MOESM3_ESM.pdf]

Description of Additional Supplementary Files

File name: Supplementary Data 1
Description: Metabolites associated with cancer cachexia.

File name: Supplementary Data 2
Description: Candidate metabolites and concentration.

File name: Supplementary Data 3
Description: Altered genes of cancer.

File name: Supplementary Data 4
Description: Metabolism related oncogenes.

File name: Supplementary Data 5
Description: Gender, age, stage, primary tumor, BMI, weight change in the last 6 months, idh1 R132H mutation and serum D2HG (ng/ml) of cancer and cancer patients.

File name: Supplementary Data 6
Description: IDH1 alteration frequency.

File name: Supplementary Data 7
Description: D2HG induced gene alteration.

File name: Supplementary Data 8
Description: D2HG induced metabolites alteration in two sets of the experiment, and the common altered metabolites.

File name: Supplementary Data 9
Description: D2HG induced gene alteration in two sets of the experiment, and the common altered genes.

File name: Supplementary Data 10
Description: Significantly altered genes and metabolites of D2hgdh.

File name: Supplementary Data 11
Description: Significantly altered genes and metabolites of D2hgdh+D2HG vs. D2hgdh.

File name: Supplementary Data 12
Description: Significantly altered genes and metabolites of D2hghd + D2HG vs. D2HG.

File name: Supplementary Data 13
Description: Shared enriched and depleted genes of D2hgdh.

File name: Supplementary Data 14
Description: Shared enriched and depleted metabolites of D2hgdh.

File name: Supplementary Data 15
Description: Primers and oligos.

File name: Supplementary Data 16
Description: Metabolites and testing parameters.

File name: Supplementary Data 17
Description: All source data underlying the graphs and charts presented in the main figures.
