# Supplementary material for: Oncometabolite D-2-hydroxyglutarate—dependent metabolic reprogramming induces skeletal muscle atrophy during cancer cachexia
Source: Commun Biol. 2023 Sep 23;6:977. doi: 10.1038/s42003-023-05366-0 (PMC10518016; doi:10.1038/s42003-023-05366-0)
Supplement: Supplementary file 6 — Reporting Summary [file 42003_2023_5366_MOESM6_ESM.pdf]

nature portfolio

Corresponding author(s): Quanjun Yang and Cheng Guo

Last updated by author(s): Sep 4, 2023

Reporting Summary

Nature Portfolio wishes to improve the reproducibility of the work that we publish. This form provides structure for consistency and transparency in reporting. For further information on Nature Portfolio policies, see our Editorial Policies and the Editorial Policy Checklist.

Statistics

For all statistical analyses, confirm that the following items are present in the figure legend, table legend, main text, or Methods section.

n/a	Confirmed	
☐	☒	The exact sample size (*n*) for each experimental group/condition, given as a discrete number and unit of measurement
☐	☒	A statement on whether measurements were taken from distinct samples or whether the same sample was measured repeatedly
☐	☒	The statistical test(s) used AND whether they are one- or two-sided<br>*Only common tests should be described solely by name; describe more complex techniques in the Methods section.*
☐	☒	A description of all covariates tested
☐	☒	A description of any assumptions or corrections, such as tests of normality and adjustment for multiple comparisons
☐	☒	A full description of the statistical parameters including central tendency (e.g. means) or other basic estimates (e.g. regression coefficient) AND variation (e.g. standard deviation) or associated estimates of uncertainty (e.g. confidence intervals)
☐	☒	For null hypothesis testing, the test statistic (e.g. *F*, *t*, *r*) with confidence intervals, effect sizes, degrees of freedom and *P* value noted<br>*Give P values as exact values whenever suitable.*
☒	☐	For Bayesian analysis, information on the choice of priors and Markov chain Monte Carlo settings
☐	☒	For hierarchical and complex designs, identification of the appropriate level for tests and full reporting of outcomes
☒	☐	Estimates of effect sizes (e.g. Cohen's *d*, Pearson's *r*), indicating how they were calculated

Our web collection on statistics for biologists contains articles on many of the points above.

Software and code

Policy information about availability of computer code

Data collection	All the data analysed in the paper are presented in the paper and the supplementary materials. The RNA-seq data have been deposited in the NCBI Sequence Read Archive under accession numbers: SUB8925446 and SUB8927518. Additional data associated with this paper are available from the authors.
Data analysis	mRNA-seq data analysis: Raw FASTQ files from RNA sequencing were analysed for transcription quantification using QianTang Biotech Co., Ltd (Suzhou, China). Transcriptome references were obtained from Ensembl. To estimate transcript abundances, HISAT was applied to the aligned reads and summarized transcript abundances into gene-level expression levels. StringTie was used for transcript prediction, and Bowtie2 was used to align sequencing reads to long reference sequences. Packages of DEseq2, EBseq, NOIseq, and PossionDis packages were used to identify up- and down-regulated genes. KEGG and GO analyses were performed using Cluster Profiler. GSEA was performed using the Java application from the Broad Institute. The full gene set from the differential gene expression analysis was ranked by "beta" value and then used as an input for GSEA pre-ranked analysis with database reference C5 Gene Ontology - Biological Process (GO-BP). Visualisation, including volcano plots, bar charts, and Venn diagrams, was performed using the standard R packages ggplot2. Enriched and depleted genes from the differential gene expression analysis were defined with an adjusted p-value cut-off of 0.05 and a fold change of 2.0.<br>Metabolomics data analysis: Multiple reaction monitoring was used for the qualitative and quantitative analysis of purified standards (Sigma, St. Louis, MO, USA). The features of the spectra were extracted using Agilent Mass Hunter Qualitative Analysis software (version B 6.0.633.0). Each peak was checked, and the abundances of all metabolites were exported. Retention times of the standards were confirmed (Table S16). Three normalization procedures, such as normalization by sum, log transformation, and auto-scaling, were used to compare individual features. The distance measure was set to Euclidean, and the clustering algorithm to Ward. Finally, the metabolic flowchart functions were constructed using Pathvisio v3.3.0 based on the KEGG database. The integrated analysis of the altered metabolites and genes was performed with the Joint Pathway analysis module of MetaboAnalyst 5.0. Volcano plots were used to filter metabolites of interest with significant fold changes at 2.0 and statistical significance at 0.05 using the software MetaboAnalyst 5.0.

Multi-omics analysis: The differential expressed genes from the RNA sequencing analysis and differential represented metabolites were entered into the common pathway analysis using the MetaboAnalyst Portal. Default parameters were used, with hypergeometric test for enrichment analysis, degree centrality for topology analysis, and gene-metabolite pathways for pathway databases. Pathways were considered statistically significant when p-values were <0.05.

For manuscripts utilizing custom algorithms or software that are central to the research but not yet described in published literature, software must be made available to editors and reviewers. We strongly encourage code deposition in a community repository (e.g. GitHub). See the Nature Portfolio guidelines for submitting code & software for further information.

Data

Policy information about availability of data

All manuscripts must include a data availability statement. This statement should provide the following information, where applicable:

- Accession codes, unique identifiers, or web links for publicly available datasets
- A description of any restrictions on data availability
- For clinical datasets or third party data, please ensure that the statement adheres to our policy

Additional data associated with this paper are available from the authors. All the data analysed in the paper are presented in the paper and the supplementary materials. The RNA-seq data have been deposited in the NCBI Sequence Read Archive under accession numbers: SUB8925446 and SUB8927518. The experiment data or analysis are included in the published manuscript and supplementary information.

Research involving human participants, their data, or biological material

Policy information about studies with human participants or human data. See also policy information about sex, gender (identity/presentation), and sexual orientation and race, ethnicity and racism.

Reporting on sex and gender	Both male and female individuals donated samples for genotyping testing. We did not perform sex-based analysis as the genetic profile of IDH1 and cancer cachexia is not expected to be shaped by sex.
Reporting on race, ethnicity, or other socially relevant groupings	All the human research was only used for IDH1 genotype analysis and metabolite D2HG detection. No race, ethnicity, or other socially relevant groupings were included for the research.
Population characteristics	Cancer patients with and without cachexia were recruited based on international consensus diagnostic criteria. Cancer patients with cachexia were included according to the following criteria: weight loss >5% in the last 6 months, or weight loss >2% in the last 6 months, and a body mass index (BMI) <20 kg/m2. Weight-stable cancer patients were those with a BMI <25 kg/m2 but no significant weight change in the previous year. Age, height, weight, cancer biomarkers, and biochemical biomarkers were obtained from patients' laboratory reports, either from either the date of diagnosis or the date closest to diagnosis. To exclude the effect of chemotherapy on the production of D2HG, the cachectic and weight-stable cancer patients were free of chemotherapy for at least 21 days at the time of samples collection. Exclusion criteria were: renal or hepatic failure; acquired immunodeficiency syndrome; inflammatory bowel disease; systemic infection.
Recruitment	Cancer patients with and without cachexia were recruited based on international consensus diagnostic criteria. Cancer patients with cachexia were included according to the following criteria: weight loss >5% in the last 6 months, or weight loss >2% in the last 6 months, and a body mass index (BMI) <20 kg/m2. Weight-stable cancer patients were those with a BMI <25 kg/m2 but no significant weight change in the previous year. Age, height, weight, cancer biomarkers, and biochemical biomarkers were obtained from patients' laboratory reports, either from either the date of diagnosis or the date closest to diagnosis. To exclude the effect of chemotherapy on the production of D2HG, the cachectic and weight-stable cancer patients were free of chemotherapy for at least 21 days at the time of samples collection. Exclusion criteria were: renal or hepatic failure; acquired immunodeficiency syndrome; inflammatory bowel disease; systemic infection.
Ethics oversight	Ethical approval was obtained from the Health Research Ethics Board of Shanghai Jiao Tong University Affiliated Sixth People's Hospital. Patients were recruited between July 2013 and May 2020. Written informed consent was obtained from all participants. Sample identification numbers were used as unique, anonymous identifiers that were independent of each patient's true identifiers. All the human research was only used for IDH1 genotype analysis and metabolite D2HG detection.

Note that full information on the approval of the study protocol must also be provided in the manuscript.

Field-specific reporting

Please select the one below that is the best fit for your research. If you are not sure, read the appropriate sections before making your selection.

☒ Life sciences ☐ Behavioural & social sciences ☐ Ecological, evolutionary & environmental sciences

For a reference copy of the document with all sections, see nature.com/documents/nr-reporting-summary-flat.pdf

Life sciences study design

All studies must disclose on these points even when the disclosure is negative.

Sample size	The numbers in each figure have been included in the methods and figure legends.

Data exclusions	We did not exclude any data from the manuscript.
Replication	Each group had at least three biological replicates, and the data are included in the methods and figure legends.
Randomization	All the male BALB/c mice (approximately 7 weeks old) and C57BL/6J mice (approximately 7 weeks old) were obtained from Shanghai SLAC Laboratory Animal Co. Ltd (Shanghai, China), with similar body weights. The randomization for grouping (10 mice per group, 5 mice per cage) was performed according to the initial undifferentiated weight.
Blinding	Not relevant to this study.

Reporting for specific materials, systems and methods

We require information from authors about some types of materials, experimental systems and methods used in many studies. Here, indicate whether each material, system or method listed is relevant to your study. If you are not sure if a list item applies to your research, read the appropriate section before selecting a response.

Materials & experimental systems

n/a	Involved in the study
☐	☒ Antibodies
☐	☒ Eukaryotic cell lines
☒	☐ Palaeontology and archaeology
☐	☒ Animals and other organisms
☐	☒ Clinical data
☒	☐ Dual use research of concern
☒	☐ Plants

Methods

n/a	Involved in the study
☒	☐ ChIP-seq
☒	☐ Flow cytometry
☒	☐ MRI-based neuroimaging

Antibodies

Antibodies used	Antibodies were from: anti-beta-actin antibody (AA128, Beyotime), anti-ADH7 antibody (ab186408, Abcam), anti-DHRS3 antibody (NBP1-80846, Novusbio), anti-GAPDH antibody (AF0006, Beyotime), anti-HMGCR antibody (ab174830, Abcam), anti-HSD17B7 antibody (16925-1-AP, Proteintech), anti-IDH1 antibody (ab172964, Abcam), anti-IDH1 antibody (ab230949, Abcam), anti-IDH1 antibody (ab113232, Abcam), anti-IDH1 (R132H) antibody (ab198123, Abcam), anti-IDH1 (R132H) antibody (DIA-H09, Dianovo), anti-IDH1 (R132H) antibody (SAB4200548, Sigma), anti-MYH antibody (sc-376157, SCBT), anti-MYH1/2/3 antibody (sc-53092, SCBT), and anti-skeletal muscle antibody (sc-32733, SCBT). Antibodies against ubiquitin (E6K4Y, #20326, 1:1500), mTOR (7C10, #2983, 1:1000), p-mTOR (Ser2481, #2974, 1:1000), p70S6 (E8K6T, #34475, 1:1000), p-p70S6 kinase (Thr389, D5U1O, #97596, 1:1000), 4E-BP1 (53H11, #9644, 1:1000), and p-4E-BP1 (Thr37/46, 236B4, #2855, 1:1000) were purchased from Cell Signaling Technology (USA). MuRF1 (C-11, sc-398608, 1:800) and MAFbx (F-9, sc-166806, 1:1000) were purchased from Santa Cruz Biotechnology (USA).
Validation	All The antibodies were validated by Western Blot.

Eukaryotic cell lines

Policy information about cell lines and Sex and Gender in Research

Cell line source(s)	Cell CT26.wt colon carcinoma cell (CRL-2638) was from ATCC. GL261 glioma cells, HEK293 (GNHu 43) and C2C12 murine myoblast (SCSP-505) were from the Cell Bank of the Typical Culture Preservation Committee of the Chinese Academy of Sciences.
Authentication	The used cell line is regularly checked.
Mycoplasma contamination	All the cell lines tested negative for mycoplasma contamination.
Commonly misidentified lines (See ICLAC register)	N/A

Animals and other research organisms

Policy information about studies involving animals; ARRIVE guidelines recommended for reporting animal research, and Sex and Gender in Research

Laboratory animals	Male C57BL/6J mice (approximately 7 weeks old) were obtained from Shanghai SLAC Laboratory Animal Co. Ltd (Shanghai, China). Male BALB/c mice (approximately 7 weeks old) were obtained from Shanghai SLAC Laboratory Animal Co. Ltd (Shanghai, China).
Wild animals	No

Reporting on sex	No obvious differences were found in the results of IDH1 mutation and occurence of cancer cachexia. We used male mice for experiments.
Field-collected samples	The study did not involve samples from the field.
Ethics oversight	All animal studies were approved by the Institutional Animal Welfare Committee at Shanghai Jiao Tong University Affiliated Sixth People Hospital in according with the government guidelines for animal manipulation in China (No.: 2021-0361).

Note that full information on the approval of the study protocol must also be provided in the manuscript.

Clinical data

Policy information about clinical studies

All manuscripts should comply with the ICMJE guidelines for publication of clinical research and a completed CONSORT checklist must be included with all submissions.

Clinical trial registration	ChiCTR-DDD-17013590
Study protocol	https://www.chictr.org.cn/showproj.html?proj=21503
Data collection	Age, height, weight, cancer biomarkers, and biochemical biomarkers were obtained from patients' laboratory reports, either from either the date of diagnosis or the date closest to diagnosis.
Outcomes	All the human research was only used for IDH1 genotype analysis and metabolite D2HG detection.
